# Mitochondrial structure and function in OCRL depleted cells

**DOI:** 10.3389/fcell.2025.1679675

**Published:** 2025-11-20

**Authors:** Ron George Philip, Priyanka Bhatia, Yojet Sharma, Padinjat Raghu

**Affiliations:** 1 Centre for Doctoral Studies, Manipal Academy of Higher Education, Manipal, India; 2 National Centre for Biological Sciences-TIFR, Bangalore, India

**Keywords:** Lowe syndrome, iPSC, mitochondria, neural stem cells, neurons, glia, metabolism

## Abstract

Lowe syndrome (LS) is an X-linked, recessive disease with a characteristic clinical triad of eye, brain, and kidney defects. LS results from mutations in the *OCRL* gene that encodes for inositol polyphosphate 5-phosphatase enzyme. The OCRL protein has been localized to multiple subcellular organelles including the plasma membrane and endo-lysosomal system, but the relevance of these to disease phenotypes is unclear. Previous studies have reported severe hypotonia at birth in LS patients along with structural changes in the mitochondria in muscle biopsies. These mitochondrial changes have been proposed to be secondary to renal tubular acidosis seen in LS patients. In this study, we find that neural stem cells and neurons differentiated from OCRL-depleted induced pluripotent stem cells (iPSCs) show mild defects in mitochondrial structure and function, whereas such defects are not seen in the iPSCs themselves. These mitochondrial phenotypes in neural stem cells and neurons were associated with modest changes in the mitochondrial transcriptome. Overall, our results indicate that loss of OCRL leads to mild cell autonomous defects in mitochondrial structure and function that is cell type-dependent.

## Introduction

The oculo-cerebro-renal syndrome of Lowe, also known as Lowe syndrome (LS), is a rare, autosomal recessive genetic disorder characterized by the clinical triad of eye, brain, and kidney abnormalities. Most patients are born with congenital cataracts, which can be surgically corrected within the first few months of infancy, and some also develop glaucoma. The nervous system and renal symptoms can vary among patients. Clinical features include intellectual disability, developmental delay, growth delay, hypotonia, and seizures. Abnormal renal function manifests as a proximal renal tubule dysfunction that worsens over time and can lead to renal failure (LOWE et al., 1952; Schoen, 1959; Sagel et al., 1970). The prevalence of LS is estimated to be approximately 1 in 500,000 individuals worldwide (Loi, 2006).

LS is a monogenic disorder caused by mutations in the *OCRL* gene, which encodes the inositol polyphosphate 5-phosphatase enzyme, OCRL. The OCRL protein predominantly dephosphorylates phosphatidylinositol 4,5 bisphosphate [PI(4,5)P_2_] to phosphatidylinositol 4 phosphate [PI4P](Attree et al., 1992; Olivos-Glander et al., 1995). OCRL also shows activity toward other polyphosphates, including [PI(3,4,5)P_3_]. OCRL is a multi-domain enzyme with a catalytic 5-phosphatase domain, an N-terminal, inactive PH domain, a RhoGAP, and an ASH domain toward the C-terminal. These domains render domain-specific functions, broadly placing OCRL in pathways regulating endo-lysosomal and actin cytoskeletal homeostasis [reviewed in (Mehta et al., 2014)]. In cells, OCRL has been localized to multiple subcellular locations, including the plasma membrane, Golgi apparatus, the endo-lysosomal system, and the primary cilia (Mehta et al., 2014).

There are a few reports of patients presenting with clinical features of renal tubular acidosis, which, on further investigation by electron microscopy, exhibited swollen mitochondria and disruption of mitochondrial cristae in renal tubular cells isolated from patients. These patients were later diagnosed to have LS, and the significance of the abnormal mitochondria was ascribed to a mere association (Schoen, 1959; Sagel et al., 1970). To ascertain the relationship of the mitochondria in LS, isolated mitochondria from muscle biopsies of an LS patient were analyzed using biochemical tests of respiration. These results indicated defective oxidative phosphorylation in the isolated mitochondria (Gobernado et al., 1984). The clinical presentation of LS was thereby associated with having some characteristics like mitochondrial disorders. Patients initially suspected to have a mitochondrial disease from clinical evaluation were later found to have an OCRL mutation from whole-exome sequencing, while having no mutations indicative of a mitochondrial disorder, highlighting the potential link between LS and mitochondrial function (Craigen et al., 2013; Dumic et al., 2020).

To date, no study has thoroughly addressed the pathophysiological basis of the mitochondrial defects detected in assays of clinical samples from LS patients. A recent study of induced neurons (iN) generated from induced pluripotent stem cells derived from LS patients and an OCRL^KO^ line showed defective oxidative phosphorylation along with elevated levels of 8-oxo-Dg, a cellular ROS indicator (Lo et al., 2024). While the defective oxidative phosphorylation in LS-derived neural cells recapitulates reports of defective mitochondrial function in LS patient-derived renal and muscle samples, the state of mitochondria in these cells is not known, and the levels of 8-oxo-DG, reported to be elevated in this study, do not directly correlate to mitochondrial ROS species.

The mitochondria are autonomous organelles that can modulate their structure and function in response to changes in the intracellular environment. One notable feature of these changes is their ability to undergo fission and fusion reactions. Fusion enables the complementation of defective mitochondria with healthy ones, while fission promotes the elimination of sub-optimally performing components through mitophagy. This dynamic process allows the mitochondria to adopt various morphologies, ranging from networked to fragmented structures. Each morphological subtype signifies a change in the stable homeostasis of the fission/fusion balance (Youle and van der Bliek, 2012). There is limited evidence for the presence of phosphatidylinositol (PI) on the mitochondrial membrane or for the mitochondria as a primary site for the synthesis of phosphoinositides. However, the mitochondria do form membrane contact sites with the endoplasmic reticulum, the primary site of PI synthesis, and it has been suggested that phosphoinositides may be transferred to the mitochondria following synthesis on other organelle membranes [reviewed in (Lourdes et al., 2024)]. This may result in secondary or indirect effects on mitochondrial structure and function in cells with altered phosphoinositide metabolism and may also explain the mitochondrial defects reported in LS patient-derived samples (see above). As many mitochondrial disorders are associated with dysfunction of the central nervous system (Pei and Wallace, 2018), we used CRISPR-Cas9-mediated OCRL knockout (OCRL^KO^) in iPSC-derived neural stem cells (NSCs) and neurons to characterize mitochondrial structure and function in OCRL-deleted cells. We found mild defects in mitochondrial structure and function in OCRL^KO^ NSC and neurons, but not in iPSCs, that likely arise from changes secondary to defects in other organelles where OCRL is localized.

## Materials and methods

### Cell lines and culture conditions

#### hiPSC

The generation and characterization of the NCRM5 hiPSCs, which were used as the control, have previously been described (Baghbaderani et al., 2015). The hiPSCs were cultured on hESC-qualified Matrigel-coated plates (Corning, # 354277) and maintained in Stemflex medium (Gibco, #A3349401) at 37 °C and 5% CO_2_ throughout. Routine passaging was done with EDTA (Sigma, #E8008), and the cultures were screened for *mycoplasma* contamination using the Lonza *Mycoplasma* detection kit (#LT-07 318).

### Generation of OCRL^KO^ iPSC

The generation and characterization of the OCRL Knockout iPSC line (OCRL^KO^) have previously been described (Sharma et al., 2024). In brief, the iPSC line was generated using CRISPR-Cas9 gene editing in the NCRM5 iPSC line, using two sgRNAs, OCRL-688-G1 and OCRL-688-G2, which were designed to target OCRL exon 8 to result in a null mutation, truncating the protein before the catalytic domain.

### Karyotyping

Chromosomal integrity of hiPSCs and NSCs was confirmed using karyotyping. For metaphase preparation, cells were arrested in the log phase by treating with 0.1 μg/mL Colcemid™ (Gibco, #15212-012) for 45 min at 37 °C. Cells were harvested in fresh Carnoy’s fixative (methanol: glacial acetic acid at 3:1), and G-banding karyotype analysis was performed at a National Accreditation Board for Testing and Calibration Laboratories, India (NABL) accredited facility.

### Generation of NSC

NSCs were generated from the NCRM5 and OCRL^KO^ iPSC lines as previously described (Akhtar et al., 2022) with a few modifications. Briefly, the iPSCs were cultured in suspension to form embryoid bodies (EB) in E6 medium. Once healthy EBs were formed, 10 μM SB (SB431542 hydrate, Sigma**,** 301,836-41-9) and 100 nM LDN (LDN 193189 Hydrochloride, Sigma, 1062368-24-4) were added to E6 and maintained for 5 days. Following this, the EBs were transferred to Neural Induction Media (NIM) with 10 μM SB and 100 nM LDN and maintained for another 5 days. The EBs were then plated onto hESC-Matrigel-coated plates (Corning, # 354277). Primary rosettes formed were then manually cut under a dissection microscope to obtain secondary rosettes. These were further purified by selection to obtain tertiary rosettes. They were then cultured as a monolayer of NSCs in Neural Expansion Medium (NEM). The NSCs were subsequently purified for enrichment and to remove neural crest cells (NCCs) using FACS (Cheng et al., 2017; Mukherjee et al., 2019). The NSCs were enzymatically dissociated using Stempro Accutase (Gibco, #A11105-01), pelleted down, and washed in PBS, following which they were immunolabeled using CD133-PE conjugated primary antibody (Abcam, #ab253271) at 10 μL antibody/million cells and CD271-AF647 conjugated antibody (Proteogen, #560326) at 15 μL antibody/million cells for 30 min at room temperature in the dark. Cells were then washed with PBS and resuspended in sorting media [(1XDMEM/F12-without phenol red (Gibco, #21041025), 1% FBS (Gibco, #16000-044)+Penicillin–Streptomycin (Gibco, #15140-122)] at a concentration of 2 million cells/mL sorting media to obtain efficient sorting. The cells were FACS-sorted using the Aria-III instrument (BD Biosciences). Forward and side scatter parameters were adjusted to eliminate cell clumps and debris. CD133+ and CD271- cells were sorted. Cells with the highest fluorescence intensity were collected and plated on Matrigel at a concentration of 0.5 million cells per well of a 12-well tissue culture plate in NEM and expanded. Cells were periodically checked for bacterial or *mycoplasma* contamination. NSCs were further characterized by immunofluorescence and neuronal differentiation.

### Generation of terminally differentiated neurons

Terminal differentiation of NSCs into cortical neurons was done as previously described (Sharma et al., 2020). Briefly, ∼30,000-50,000 NSCs were plated in confocal dishes coated with poly-L-ornithine/laminin and maintained in neural differentiation medium (NDM) supplemented with 10 ng/mL BDNF (Gibco # PHC7074), GDNF (Gibco #PHC7041), IGF (Gibco, #PHG0078) ascorbic acid (50 μM, Sigma # A4544), and dbcAMP (1000μM, Sigma #D0627). During the first 14 days after plating, cells were treated with 2 μM DAPT (Sigma-Aldrich #D5942) to synchronize the neuronal maturation process. The neurons were differentiated until days *in vitro* (DIV) 30 and 40.

### Mitochondrial morphology and network characteristics

The morphology of the mitochondria was quantified by live cell staining with MitoTracker™ Green FM (Invitrogen™, #M7514). iPSCs/NSCs were seeded at 1 × 10^5^ cells/dish in confocal dishes (Biofil, #BDD011035) and kept overnight. The following day, the cells (iPSCs, NSCs) were incubated with 20 nM MitoTracker™ Green FM for 30 min at 37 °C, followed by washing once with PBS. Then, 1 μg/mL Hoechst (Invitrogen™, #33342) was added to the cells for the last 10 min of the MitoTracker™ Green FM incubation window to stain the nucleus. The cells were then immediately analyzed on the Olympus FV3000 confocal microscope with a 60X oil immersion objective. Hoechst was excited using the 405 laser, and emission bands were collected at 460 nm. MitoTracker™ Green FM was excited with the 488 laser and data collected at the ∼515 nm range. For each genotype, 3–4 dishes were imaged, with 5–6 images collected per dish and approximately 15–20 cells per image. Images were analyzed on ImageJ (National Institute of Health, USA, http://imagej.nih.gov/ij). A Z-stack (maximum intensity projection) of each image was taken, and ROIs were drawn to mark the cell boundary per cell from the background signal of the MTG channel. Images were then cropped using the ROIs to obtain images of each cell. These single-cell images were then analyzed using the Mitochondria Analyser Plugin, and thresholding and contrast optimization were adjusted automatically. Parameters of mitochondrial morphology and network characteristics were then obtained from the plugin analysis.

### Mitochondrial membrane potential (MMP)

#### Imaging measurement

Mitochondrial membrane potential was measured using tetramethylrhodamine methyl ester (TMRM) (Invitrogen™, #T668). Briefly, the cells (iPSCs, NSCs) were seeded at 1 × 10^5^ cells/dish in confocal dishes (Biofil, #BDD011035) and kept overnight. The following day, they were incubated with 20 nM TMRM for 30 min at 37 °C. Then, 1 μg/mL Hoechst (Invitrogen™, #33342) was added to the cells for the last 10 min of incubation. The cells were then washed once with PBS and immediately analyzed on the Olympus FV3000 confocal microscope with a 60X oil immersion objective. Hoechst was excited using the 405 laser, and emission bands collected at 460 nm. TMRM was excited with the 561 laser, and data were collected at ∼575 nm. For each genotype, 3–4 dishes were imaged, with 5–6 images collected per dish and approximately 15–20 cells per image. Images were analyzed on ImageJ (National Institute of Health, USA, http://imagej.nih.gov/ij). A z-stack of the images was taken for analysis. ROIs of equal area were drawn over pools of the mitochondria in the TMRM channel. Mean Fluorescence Intensity Units (MFU) per ROI was then used for measurements. For measurement of MMP in neurons, NSCs were differentiated to neurons at DIV30, DIV40 in confocal dishes and subsequently stained with 20 nM TMRM for 30 min at 37 °C and 1 μg/mL Hoechst for the last 10 min, following which they were immediately taken for analysis on confocal microscopy. MFU per ROI drawn across pools of the mitochondria were analyzed on ImageJ, similar to the analysis of NSC, iPSC.

#### Flow cytometry measurement

For the flow cytometry-based analysis, the cells were initially dissociated using EDTA (Sigma, #E8008) for iPSCs and Stempro Accutase (Gibco, #A11105-01) for NSCs, following which they were pelleted down and resuspended in TMRM at 20 nM concentration in either Stemflex or NEM media, respectively, for 30 min at 37 °C. FCCP (10 μM) was added as a control to the respective cells for the last 5 min. Cells were then washed with PBS once and resuspended in PBS at two million cells/mL and transferred to FACS tubes for analysis on the BD Fortessa instrument. Forward and side scatter parameters were adjusted to remove cell debris and clumps. TMRM was excited using the 561 laser and fluorescence intensity collected at the ∼575 nm range. Gating was used to mark the fluorescence intensity range of the control cells. Comparatively, the percentage of cells falling within the set gate was measured for the OCRL^KO^ cells. Two biological replicates were kept for each genotype under each condition. Approximately 10,000 cells were measured from each replicate.

#### Seahorse assay measurement of cellular respiration

Mitochondrial stress assays, oxygen consumption rate (OCRs), and glycolytic stress assay (extracellular acidification rate, ECAR) were measured using the Seahorse XFe24 Extracellular Flux analyzer (Agilent). The drug cartridges were kept for overnight incubation in XF Calibrant solution in a non-CO_2_ incubator at 37 °C. The cartridges were taken out 1 h before the assay, and the injection ports were loaded with the adequate drugs at their appropriate concentrations. Control and OCRL^KO^ NSCs were seeded at 1 × 10^5^ cells/well in NEM at five wells/genotype and grown overnight at 37 °C with 5% CO2. One hour before measurement, the culture medium was removed and replaced by Agilent Seahorse XF Base Medium complemented with 10 mM glucose, 1 mM sodium pyruvate, and 200 mM L-glutamine, and incubated at 37 °C, without CO_2_. Basal oxygen consumption was measured three times, followed by three measurement cycles after each addition of 1.5 μM oligomycin A, 0.5 μM carbonyl cyanide 4-(trifluoromethoxy) phenylhydrazone (FCCP), and 1 μM rotenone and antimycin A. After overnight incubation of cells seeded for ECAR measurements, at five wells/genotype, the culture medium was removed and replaced with Agilent Seahorse XF Base medium complemented with 200 mM L-glutamine, and incubated at 37 °C for 1 hour before measurement. Basal ECAR was measured three times, followed by three measurement cycles after each addition of 10 mM glucose, 3 μM oligomycin, and 50 mM 2-DG. One measurement cycle consisted of 3 min of incubation and 3 min of measurement. After completion of OCR and ECAR measurements, the culture medium was removed, and RIPA buffer with a protease inhibitor was used to make protein lysates from each well for protein estimation. Total protein from each well was quantified using the Pierce™ BCA protein assay kit (Thermo Scientific™, #23227). The OCR and ECAR readings were normalized to the total protein content of each well in the Wave controller software and data exported to Excel.

#### NGS sequencing

Total RNA was extracted from well-characterized iPSCs, NSCs, and 30-day-old neurons of control and OCRL^KO^ lines using TRIzol (Ambion, Life Technologies, #5596018) according to the manufacturer’s protocol in three biological replicates. RNA was isolated using the chloroform extraction method. The RNA was quantified using a Qubit4 dsDNA HS Assay Kit (Thermo Fisher Scientific, #Q32854) and run on a Bio-analyzer chip (Agilent High Sensitivity DNA Chip, #5067-4626) to assess integrity. rRNA depletion was done using the NEBNext® rRNA Depletion Kit v2 (Human/Mouse/Rat) with RNA Sample Purification Beads (Catalog no-E7405L). NEBNext® Ultra™ II Directional RNA Library Prep with Sample Purification Beads (Catalog no-E7765L) (RIN values > 9) was used per sample for the library preparation. The libraries were then sequenced on the Illumina Novaseq 6000 sequencing platform using a 2 × 100 bp sequencing format.

#### Bioinformatics analysis

Illumina-sequenced paired-end reads were obtained from sequencing as mentioned above. The quality of processed reads (adapter removal and trimming) was evaluated using FastQC. The RNA-seq reads were then mapped onto the human reference genome (hg38) using HISAT2 (version: 2.2.1), and the resulting BAM files containing the aligned reads were provided to HTSeq (version: 2.0.9) to obtain a gene-level read count table using the reference annotation file (GTF format). Read counts data were then transformed using the regularized log(rlog) transformation method that is implemented in DESeq2. Differential gene expression comparisons were made with NCRM5 as the control, with a log2FC threshold as indicated for NSCs/neurons. Functional analysis of the differentially expressed genes (DEGs) was made from the “Biological process” terms from the GO database. For over-representation analysis (ORA), GO term enrichment was made using the *topGO* R package (version 2.52.0) (Alexa and Rahnenfuhrer, 2023). For gene set enrichment analysis, GO term enrichment was made from the “Biological process” terms from the GO database using the *clusterProfiler* R package (version 4.8.3) (Wu et al., 2021). PCA distance map and dotplots were generated using the *ggplot2* R package (version 3.5.1), and heatmaps of differential gene expression were made using the *ComplexHeatmap* R package (version 2.16.0).

#### Statistics

Appropriate t-tests were used to compare datasets of two groups. All statistical analyses were done on GraphPad Prism (version 9). Throughout the text, N = number of biological replicates; n = number of cells analyzed.

## Results

### Loss of OCRL results in mitochondrial fragmentation

**FIGURE 1:**
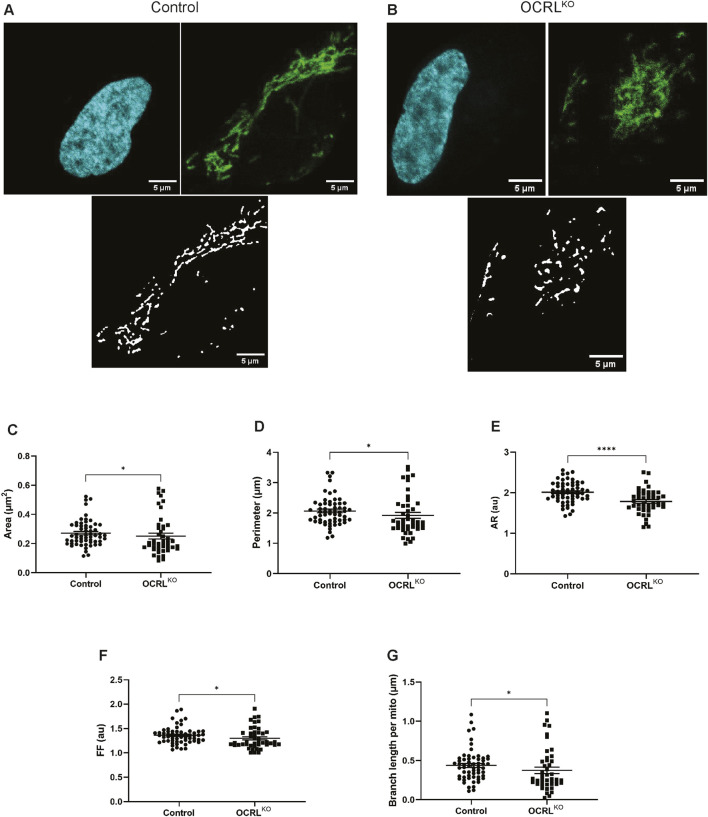
Loss of OCRL results in smaller, spherical mitochondria in NSCs: **(A–G)** Automated Image analysis via Mitochondria Analyser plugin (Fiji) characterizing the mitochondrial morphology of Control and OCRL^KO^ NSCs. Each dot represents measurements of the corresponding mitochondrial parameter from a single cell. [**A,B**] Representative z-stack maximum intensity projections of the mitochondria in single cells stained with Hoechst (cyan), MitoTracker Green (MTG) dye (green), and their corresponding segmented images post-image processing (black, white) of control **(A)** and OCRL^KO^ NSCs **(B)**. The following parameters were measured: **(C)** mean area; **(D)** mean perimeter; **(E)** aspect ratio (AR); **(F)** form factor (FF); **(G)** branch length per mitochondrion. All panels’ data represented as mean+/-SEM. Statistical significance calculated using two-tailed *Mann-Whitney U test*, *P = 0.0175; (N = 3, n∼50).

To test the potential function of OCRL in mitochondrial structure and function, we utilized iPSCs engineered to create OCRL loss-of-function (OCRL^KO^) (Sharma et al., 2024). OCRL^KO^ was generated from the control iPSC NCRM5 (https://commonfund.nih.gov/stemcells/stem-cell-lines-scl), which was used as the isogenic control (hereafter referred to as “control”). OCRL^KO^ and control iPSCs were differentiated into neural stem cells, which were then purified and characterized by immunocytochemistry (Supplementary Figure S1A). We visualized the morphology of the mitochondria in control and OCRL^KO^ NSCs through live cell staining with MitoTracker™ Green FM (MTG), which selectively accumulates in the mitochondrial matrix and covalently binds to mitochondrial proteins (Figures 1A,B). Using an automated image analysis pipeline with the mitochondria analyzer plugin on Fiji (Chaudhry et al., 2020), we screened several parameters of mitochondrial morphology on a per-cell basis. The mitochondria in the OCRL^KO^ NSC displayed a slight reduction in the area and perimeter (Figures 1C,D), which are size-related parameters, indicating smaller mitochondria. Shape parameters, including Aspect Ratio (AR) and Form Factor (FF), were also moderately diminished in the OCRL^KO^ cells compared to the control (Figures 1E,F), indicating increased sphericity. The branching of mitochondrial tubules to form a network facilitates faster dynamics and improved functionality (Viana et al., 2020; Chuphal et al., 2024; Holt et al., 2024). OCRL^KO^ cells exhibited a slightly reduced branch length per mitochondrion compared to the control (Figure 1G), suggesting a reduced mitochondrial network. Overall, our morphological characterization of the mitochondria indicates that loss of OCRL leads to moderately smaller, spherical mitochondria with comparatively reduced branching.

### Mitochondria in OCRL^KO^ NSC show reduced mitochondrial membrane potential

**FIGURE 2:**
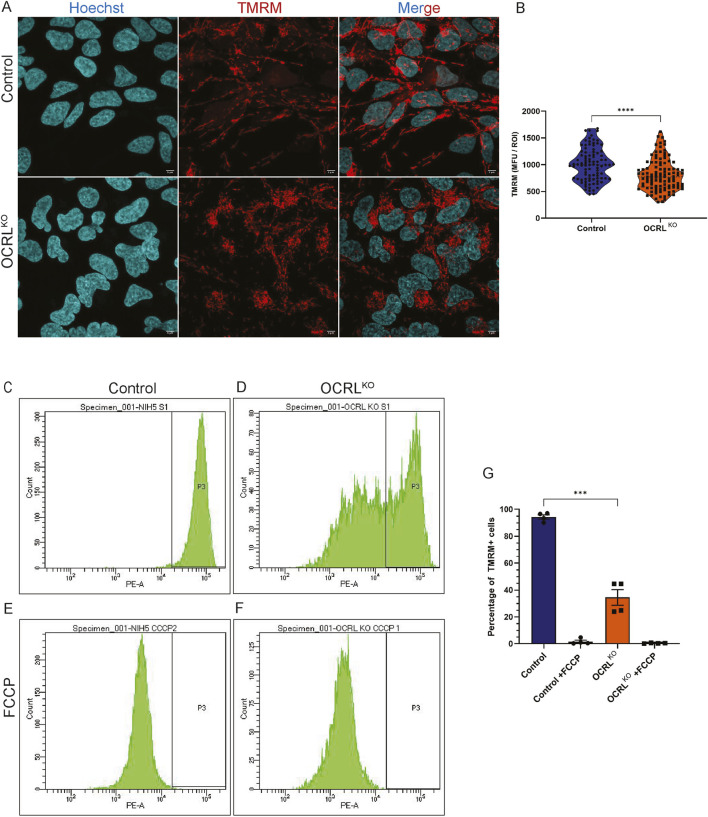
Mitochondria in OCRL^KO^ NSCs show reduced mitochondrial membrane potential: **(A,B)** Confocal microscopy imaging of TMRM. Control and OCRL^KO^ NSC cultures stained with TMRM dye (20 nM) (red) to mark polarized mitochondria and Hoechst (1 μg/mL) (cyan) to mark the nucleus. **(B)** Violin plot depicts Mean Fluorescence Units (MFU) of the TMRM signal for each genotype. Each data point corresponds to the MFU of 1 ROI drawn over a pool of mitochondria. Data represented as mean+/-SEM, ****P < 0.0001; two-tailed *Mann-Whitney U test*; (N = 3, n∼100) **(C,G)** Flow cytometry-based measurement of TMRM. **(C,D)** Histogram of TMRM signal intensity per cell of control **(C)** and OCRL^KO^ **(D)** NSCs. Gating was used over the histogram (P3) of control NSCs to mark the TMRM signal intensity of control. **(E,F)** Histogram of TMRM signal intensity per cell of FCCP (10 μM)-treated control **(E)** and OCRL^KO^ **(F)** NSCs. **(G)** Bar graph depicting the percentage of cells in the P3 gate. Each data point represents one biological replicate. Statistical significance calculated using two-tailed *paired t*-test, ***P < 0.0009; (N = 2, n = 10,000 cells/tube).

The mitochondrial membrane potential (MMP) is a crucial aspect of mitochondrial respiration, generated by components of the electron transport chain (ETC.), and is essential for ATP synthesis. Movement of electrons across the ETC is responsible for pumping H^+^ ions across the inner mitochondrial membrane; this difference in concentration of ions generates an electrochemical gradient across the membrane, known as the MMP (Nolfi-Donegan et al., 2020). A highly polarized membrane supports active oxidative phosphorylation, while a depolarized membrane leads to decreased reliance on oxidative phosphorylation, causing cells to shift toward glycolysis (Meacham et al., 2022). We measured the MMP in the OCRL^KO^ and control NSC using tetramethylrhodamine methyl ester (TMRM), a lipophilic cationic dye that accumulates in the mitochondrial matrix in proportion to the membrane potential. NSCs were stained with TMRM, Hoechst was used to mark the nucleus, and cells were imaged using confocal microscopy (Figure 2A). Regions of interest (ROIs) drawn across pools of mitochondria were employed to measure the fluorescence intensity. The data showed a range of TMRM intensities in both genotypes, with cells in the OCRL^KO^ NSC culture exhibiting a modest but significant reduction in signals compared to the control (Figure 2B). To better understand the distribution of OCRL^KO^ NSC with varying MMP, we employed flow cytometry to measure the TMRM intensity. This enabled us to plot the distribution of cells with varying TMRM intensities at the single-cell level. In control NSCs, we observed a single peak of TMRM signal intensity (Figure 2C), indicating that all cells in the culture had approximately the same MMP, reflecting healthy polarized mitochondria. In contrast, OCRL^KO^ NSC displayed at least two sub-populations of cells with different TMRM signal intensities (Figure 2D). Approximately 50% of the cells showed a peak that overlapped with that of control NSC, while approximately 40%–50% of the cells exhibited a peak of lower TMRM intensity. Both control and OCRL^KO^ NSC showed a reduction in the MMP following treatment with FCCP (10 µM) (Figures 2E,F), which served as a positive control to confirm that TMRM effectively labels polarized mitochondria. Taken together, measurement of MMP using both confocal microscopy as well as flow cytometry reveals a mixture of polarized and heterogeneous depolarized mitochondria in OCRL^KO^ NSC.

### Depolarized mitochondria in OCRL^KO^ NSC affect mitochondrial function

**FIGURE 3:**
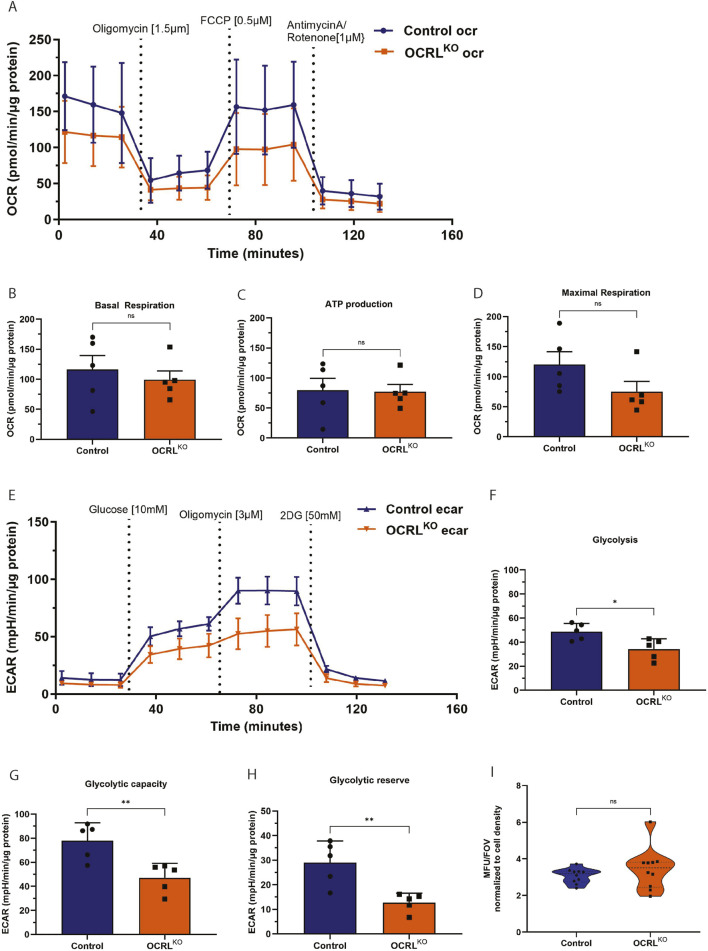
OCRL^KO^ NSCs show altered parameters of cellular respiration: (**A–H)** Seahorse assay on control and OCRL^KO^ NSCs, indicating various parameters of cellular respiration (N = 5 biological replicates). **(A–D)** Oxygen Consumption Rate (OCR) measured over various treatments, as indicated in the graph **(A)**. Basal respiration **(B)**, ATP production **(C)**, and maximal respiration **(D)** are represented as bar graphs, with grouped data points from the OCR measurements. **(E)** Extracellular Acidification Rate (ECAR) measurements over various treatments as indicated in the graph. Glycolysis **(F)**, glycolytic capacity **(G)**, and glycolytic reserve **(H)** are represented as bar graphs with grouped data points from ECAR measurements. **(I)** MitoTracker Red CM-H_2_Xros measurements of mitochondrial ROS species in Control and OCRL^KO^ NSCs (N = 2, n = 5). All panels’ data are represented as mean+/-SEM. Statistical significance calculated using two-tailed *Mann–Whitney U* test, *P < 0.05, ns > 0.05.

More than 95% of cellular energy requirements are supplied by mitochondrial oxidative phosphorylation, the principal process for ATP production. The H^+^ ions concentrated in the mitochondrial intermembrane space are pumped back into the matrix via the P0/P1 ATP synthase complex, which phosphorylates ADP to generate ATP for the cell. Oxygen consumption represents the terminal step of the oxidative phosphorylation (OXPHOS) process that produces cellular energy (Nolfi-Donegan et al., 2020). Various parameters of this cellular respiration were measured using the Seahorse Assay to assess mitochondrial ATP production (Figure 3A). We found that OCRL^KO^ NSC exhibited slightly decreased basal respiration compared to control (Figure 3B). To assess ATP production, we injected oligomycin and measured the oxygen consumption rate (OCR) and found it to be slightly reduced in OCRL^KO^ NSC (Figure 3C). The FCCP-mediated uncoupling of the proton gradient revealed that maximal respiration was also slightly lower in the OCRL^KO^ NSC (Figure 3D). Taken together, these results could indicate a defect in the OXPHOS pathway at basal levels or a shift in metabolism toward glycolysis. To distinguish between these two, we measured the extracellular acidification rate (ECAR) in these cells (Figure 3E). The OCRL^KO^ NSC demonstrated a significant reduction in glycolysis at basal levels (Figure 3F). Treatment with oligomycin at a higher concentration resulted in a decreased glycolytic capacity in OCRL^KO^ (Figure 3G). Furthermore, treatment with 2DG, a non-metabolizable glucose analog, revealed reduced glycolytic reserves in OCRL^KO^ compared to control (Figure 3H). Defects in the OXPHOS pathway have a high potential to result in elevated mitochondrial ROS levels in the cell, which can be detrimental (Okoye et al., 2023). We therefore checked the levels of ROS in OCRL^KO^ NSC using the CM-H2XROS dye. Our results indicate no significant difference in the mitochondrial ROS levels measured between control and OCRL^KO^ (Figure 3I). Overall, the results indicate modest mitochondrial function defects in OCRL^KO^ NSC.

### Changes of mitochondrial function in OCRL^KO^ NSC are independent of PI(4,5)P_2_

**FIGURE 4:**
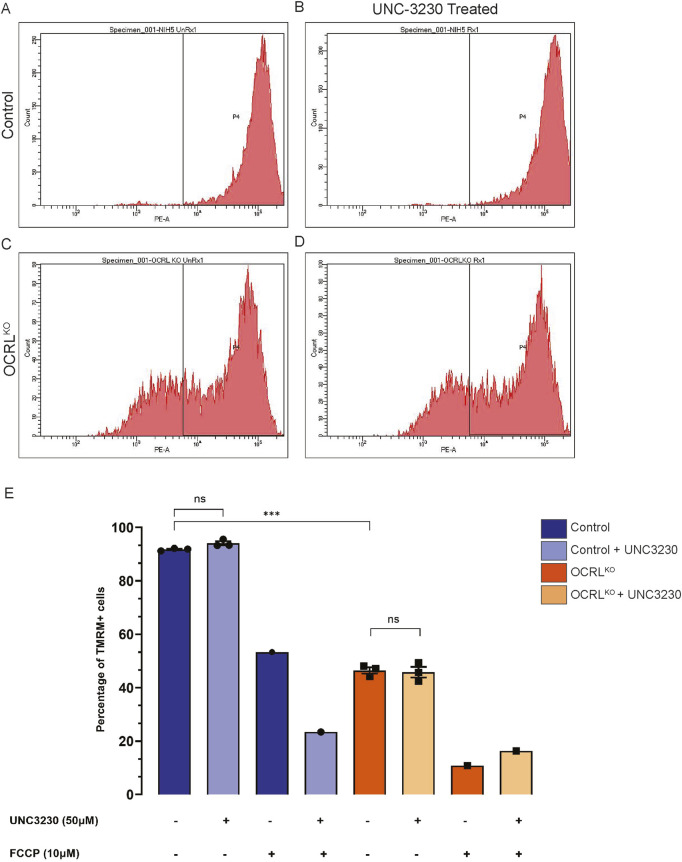
Mitochondrial changes in OCRL^KO^ NSC culture is independent of elevated PI(4,5)P_2_: **(A–E)** PIP5K inhibitor (UNC3230) treatment to reduce elevated PI(4,5)P_2_ levels. Control and OCRL^KO^ NSC cultures were treated with UNC3230 (50 μM) and vehicle control (DMSO, 0.05%) for 7 days and incubated with TMRM (20 nM) × 30 min at 37 °C, following which they were taken for flow cytometry analysis. **(A–D)** Histograms depicting TMRM signal intensity. Gating was used over the histogram (P4) of the control, untreated NSCs, to mark the TMRM signal of the control. **(E)** Bar graph indicating the percentage of cells in the P4 gate under different treatment conditions indicated in the x-axis. Legend identifies the genotype. Vehicle control (DMSO 0.05%) was added wherever marked as UNC3230 “-.” Each data point represents one biological replicate. Statistical significance calculated using unpaired t-test with Welch’s correction, N = 1, n = 3, *P = 0.0003.

We have previously shown that loss of OCRL results in elevated levels of PI(4,5)P_2_ (Akhtar et al., 2022). To test whether the changes in NSC mitochondrial function were a direct consequence of this elevation, we rescued the elevated PI(4,5)P_2_ levels in OCRL^KO^ NSC by pharmacologically inhibiting PIP5K, the enzyme responsible for synthesizing the major pool of PI(4,5)P_2_ in cells. If the elevation of PI(4,5)P_2_ leads to mitochondrial defects, reversing PI(4,5)P_2_ levels to those of controls should reverse the reduction in MMP. We used the drug UNC3230, a PIP5K inhibitor, to treat both control and OCRL^KO^ NSC cultures for 7 days. Preliminary experiments using an NSC line carrying a fluorescent reporter for plasma membrane PI(4,5)P_2_ levels were used to determine the concentration of UNC3230 that reduced the levels of PI(4,5)P_2_ (Supplementary Figure S2). Following UNC3230 treatment, we employed flow cytometry to estimate MMP using TMRM signal measurements (Figure 4). The control NSC showed no change in TMRM intensity following UNC3230 treatment compared to untreated cultures (Figure 4A,B, quantified in E). As in previous experiments, OCRL^KO^ NSC displayed two distinct populations of cells, with 50% of the cells exhibiting reduced TMRM (Figure 4C); this was not altered by prior treatment with UNC2320 (Figure 4D). FCCP treatment served as a positive control for mitochondrial TMRM staining. FCCP treatment across all conditions led to depolarization of the mitochondria with no discernible difference between control and OCRL^KO^ (Figure 4E). These results suggest that PI(4,5)P_2_ levels do not affect MMP in either wild type or OCRL^KO^.

### Mitochondrial functional changes are seen in OCRL^KO^ neurons

**FIGURE 5:**
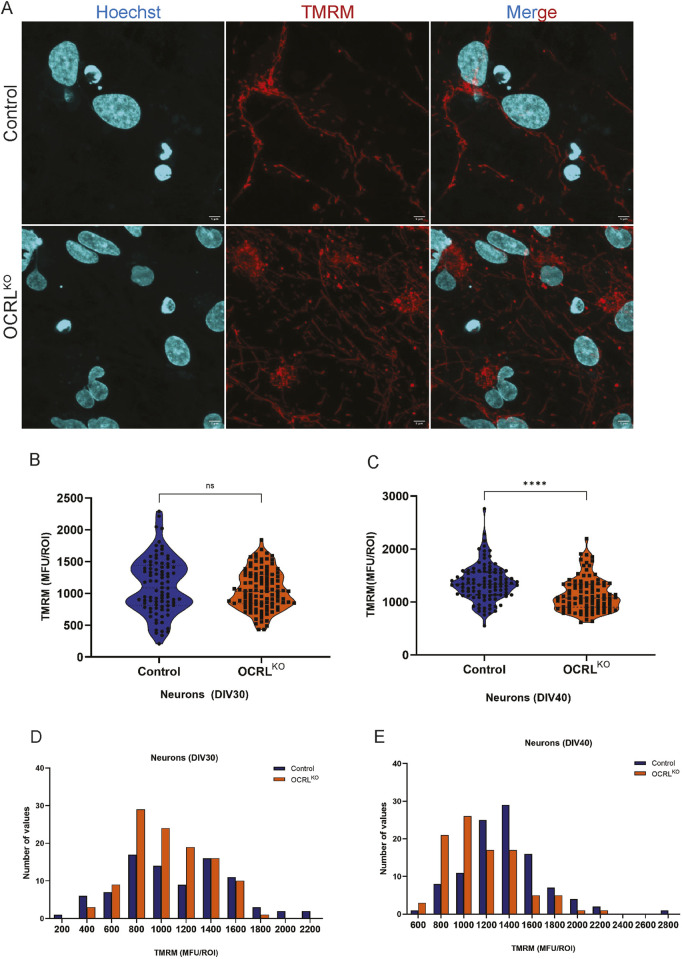
Mitochondrial changes in NSCs persist over neuronal differentiation: **(A,B)** Control (Top row) and OCRL^KO^ (bottom row) neurons (DIV30) stained with TMRM (red), Hoechst (cyan) on live imaging on a FV3000 confocal microscope. **(B,C)** Violin plot depicting TMRM signal intensity calculated as the MFU of ROIs drawn over pools of the mitochondria per genotype in control and OCRL^KO^ neuronal culture of DIV30 and DIV40, respectively. Each data point represents 1 ROI. Statistical significance calculated using the Mann–Whitney test, *P < 0.005; ****P < 0.0001, N = 3, n∼100. **(D,E)** Bar graph indicating the frequency distribution of the different TMRM intensities, calculated as the MFU of ROIs plotted in bins on the x-axis, and number of values for each bin plotted on the y-axis. Legend indicates the color of the genotype.

While NSCs are known to use both glycolysis and mitochondrial respiration, it is reported that on differentiation to neurons, they shift toward OXPHOS primarily for ATP production (Rangaraju et al., 2019; Scandella et al., 2023). To assess whether the mitochondrial changes we observed in OCRL^KO^ NSC persist in neurons, we differentiated NSC and stained the neurons (characterized in Supplementary Figure S3A) at 30 DIV and 40DIV with TMRM (Figure 5A). In NSCs, a reduced TMRM signal was the most prominent finding (Figure 2G). However, 30DIV control and OCRL^KO^ neurons displayed a wide range of TMRM intensities, which became more robust/uniform by 40DIV (Figure 5A). Recapitulating observations in NSC, there was an almost five-fold range of TMRM intensities seen in both control and OCRL^KO^ neurons at DIV30 (Figure 5B); this variability reduced by DIV40 (Figure 5C). OCRL^KO^ neurons showed a modest and nonsignificant reduction in TMRM intensity at DIV30 (Figure 5B). Analysis of a binned frequency distribution of TMRM intensities revealed a slight left shift of TMRM intensity in OCRL^KO^ mitochondria, compared to control (Figure 5D). As differentiation proceeds, by DIV40, the TMRM intensity in OCRL^KO^ neurons increased compared to that at DIV30. However, there was now a substantial reduction in OCRL^KO^, compared to control (Figure 5C); analysis of binned intensities revealed a left shift in OCRL^KO^ compared to control (Figure 5E).

### Mitochondrial MMP is not altered in OCRL^KO^ iPSC

**FIGURE 6:**
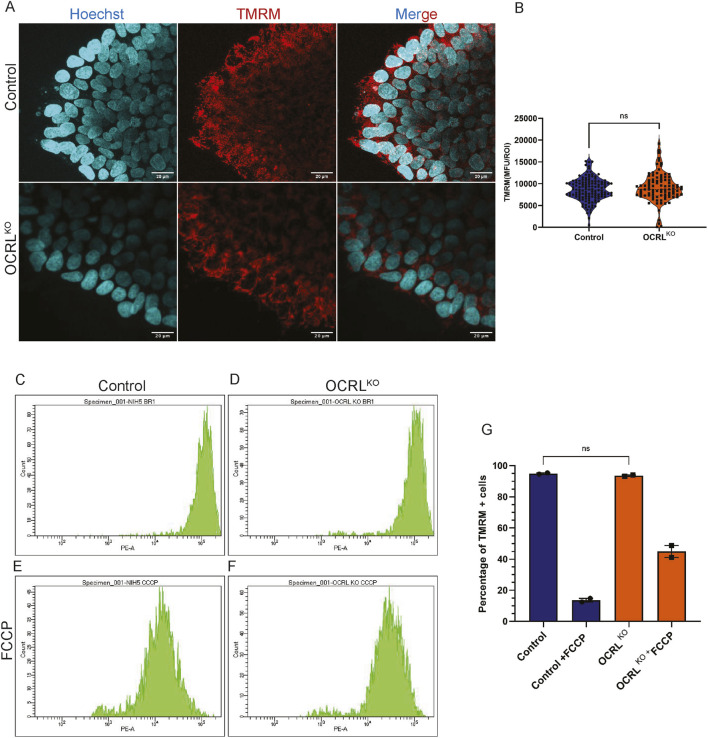
Reduction in MMP in OCRL ^KO^ is specific to tissues primarily relying on oxidative phosphorylation: **(A,B)** Confocal microscopy imaging of TMRM. Control and OCRL^KO^ iPSC cultures stained with the TMRM dye (20 nM) (red) to mark polarized mitochondria and Hoechst (1 μg/mL) (cyan) to mark the nucleus. **(B)** Violin plot depicts Mean Fluorescence Units (MFU) of the TMRM signal for each genotype. Each data point corresponds to the MFU of 1 ROI drawn over a pool of mitochondria. Data represented as mean+/-SEM, *P < 0.05, ns = 0.2993; statistical significance calculated using two-tailed *Mann–Whitney U test*, (N = 2, n∼100) **(C–G)** Flow cytometry measurement of TMRM intensity of iPSCs. **(C,D)** depicts histograms of TMRM signal intensity per cell of control and OCRL^KO^ iPSCs. **(E,F)** Control and OCRL^KO^ iPSCs treated with FCCP (10 μM). **(G)** Bar graph depicting % of TMRM + cells in the same gate as control across all conditions. Each data point represents one biological replicate. Statistical significance calculated using two-tailed paired t-test, *P = 0.3333, (n = 2 biological replicates).

Reduced MMP can be noted because of a plethora of conditions, one of which may be due to the fragmentation of the mitochondria, resulting in a limited capacity for oxidative phosphorylation (OXPHOS). Another may be due to less dependence on oxidative phosphorylation and the use of glycolysis as the main source for ATP generation. Stem cells are known to rely on glycolysis as their major source of energy and later switch to OXPHOS on cell fate specification and differentiation (Scandella et al., 2023). We measured the MMP in iPSC comparing control with OCRL^KO^ using the TMRM staining and confocal microscopy (Figure 6A). ROIs were drawn across pools of mitochondria in both genotypes to estimate MMP. Surprisingly, there was no significant difference in the TMRM intensity between iPSCs of control and OCRL^KO^ (Figure 6B). To further analyze the distribution of MMP among iPSC cells in the culture, we analyzed their MMP by flow cytometry using TMRM staining. The histograms recapitulated the results obtained with confocal imaging. Single peaks of TMRM intensity were seen in both control and OCRL^KO^ iPSC (Figures 6C,D), and no significant difference was found in the MMP between control and OCRL^KO^ (Figure 6G). After recording the baseline signal, FCCP (10 μM) was added to the cells to depolarize the mitochondria as a positive control (Figures 6E,F). Cells were analyzed by flow cytometry, and we found that both control and OCRL^KO^ showed loss of MMP on FCCP treatment (Figures 6E,F quantified in 6G); it seemed that OCRL^KO^ was less sensitive to FCCP treatment compared to control (Figure 6I). Overall, we found no significant differences in mitochondrial function between iPSC of control and OCRL^KO^.

### Impact of OCRL depletion on the mitochondrial transcriptome

**FIGURE 7:**
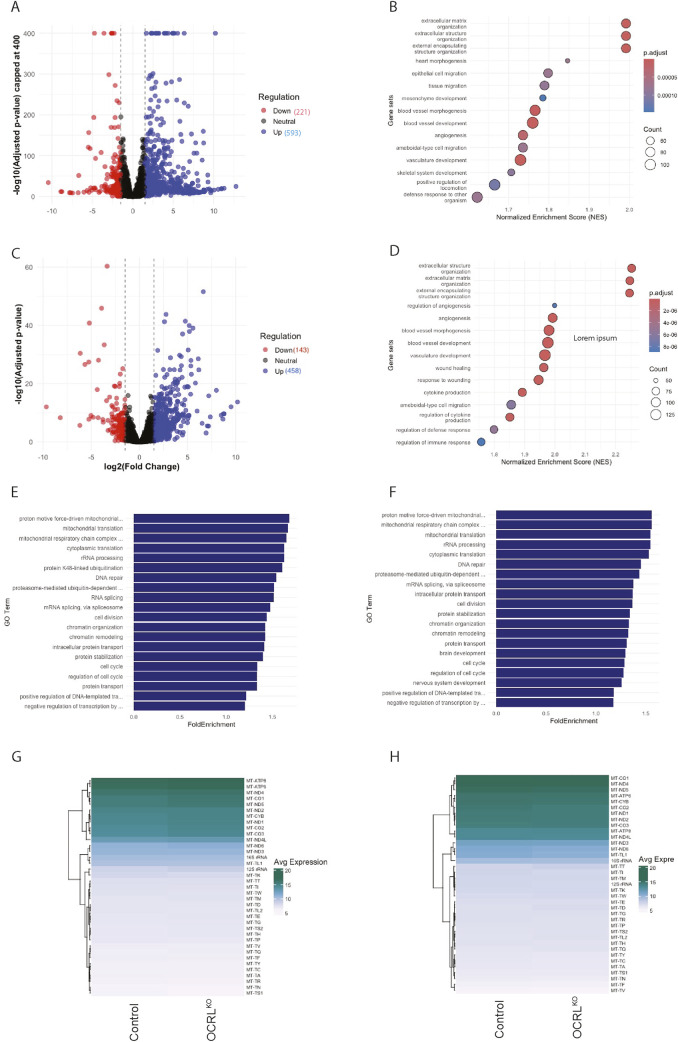
Transcriptomic analysis of OCRL^KO^ NSCs shows moderate dysregulation in mitochondrial gene expression: **(A)** Scatter plot of DEGs in OCRL^KO^ NSCs *versus* Control. 593 genes were found to be upregulated (blue dots), and 221 genes were found to be downregulated (red dots) (log_2_ fold change cut-off −1.5 to +1.5, p, p adj. value < 0.05). y-axis (-log10 adjusted p-value) capped at 400, values above cap were capped for visualization clarity. **(B)** Dot plot depicting enriched biological process GO terms on the y-axis and Normalized Enrichment Score (NES) on the x-axis calculated from gene set enrichment analysis (GSEA) for NSC, size of the dots indicates count of number of DEGs within each GO term, and shading represents adjusted p value < 0.05. **(C)** Scatter plot of DEGs in OCRL^KO^ neurons (DIV30) *versus* Control. A total of 458 genes were found to be upregulated (blue dots), and 143 genes were found to be downregulated (red dots) (log_2_ fold change cut-off −1.5 to +1.5, p, p adj. value < 0.05). **(D)** Dot plot depicting enriched biological process GO terms on the y-axis and Normalized Enrichment Score (NES) on x-axis, calculated from GSEA for neurons; size of the dots indicates the count of number of DEGs within each GO term; shading represents the adjusted p value < 0.05. **(E,F)** Bar graphs of Biological Process-enriched GO terms in Control and OCRL^KO^ NSCs and neurons (DIV30) respectively, calculated from topGO. X-axis represents the fold enrichment score, and y-axis indicates the GO term; top 20 GO terms are represented with adjusted p value <0.05. **(G,H)** Heatmaps of variance-stabilized average expression of all mitochondrial DNA-coding genes in control and OCRL^KO^ NSCs and neurons (DIV30), respectively.

To characterize changes in mitochondrial gene expression associated with the loss of OCRL, which may explain the mitochondrial functional and morphological changes seen in NSCs, we performed a transcriptomic analysis of OCRL^KO^ NSC compared to controls. We found ca. 13,941 differentially expressed genes (DEGs) in the OCRL^KO^ NSCs (Figure 7A). Gene Ontology (GO) analysis performed on a significance filtered DEG set (Figure 7B) did not reveal any terms associated with mitochondrial structure or function. Likewise, a transcriptomic analysis on DIV30 neurons differentiated from control and OCRL^KO^ NSC showed ca. 143 downregulated and 458 upregulated genes (Figure 7C); GO analysis of this DEG set also did not reveal any terms associated with mitochondrial structure and function (Figure 7D). Lastly, the average expressions of all mitochondrial DNA coding genes in control and OCRL^KO^ NSCs and neurons (DIV30) were not different (Figures 7G,H). The transcriptomic profile of both NSC and neurons were confirmed for both control and OCRL^KO^ cultures (Supplementary Figure S1B, Supplementary Figure S3B).

We also performed a GO enrichment analysis using an over-representation analysis (ORA) pipeline (topGO) with the entire list of DEGs from the NSC transcriptome, i. e., regardless of statistical significance, and the top three enriched GO terms were related to the mitochondria with a fold enrichment score of ∼1.5 (Figure 7E). We noted an enrichment of multiple mitochondria-related GO terms; this was also true of the transcriptome from DIV30 neurons (Figure 7F). This finding indicates that there is a subtle transcriptional dysregulation of mitochondrial genes as a coherent group in the OCRL^KO^ NSC that may not be individually statistically significant in ranked or Log2 fold change cut-off filtered approaches. We generated heatmaps of the individual genes that were dysregulated in the GO terms that were picked up (Supplementary Figure S4), which reflected modest changes in average gene expression seen in the ORA. Lastly, we also compared changes in the expression of genes that are encoded in the nuclear genome but localize to and impact mitochondrial function; there was no substantive change in these either (Supplementary Table S1).

## Discussion

Although previous studies have reported mitochondrial defects in cells of biopsy samples from muscle and renal tissue of LS patients, it has not been clear what the source of these defects might be. Notably, mitochondrial defects have been reported in biopsies from the muscle (Gobernado et al., 1984; Dumic et al., 2020), a tissue not typically affected in LS patients. For example, metabolic defects in the circulation secondary to impaired renal function could, in turn, lead to mitochondrial defects in tissues such as the muscle. In this study, we noted mitochondrial defects in cultured neural stem cells and neurons differentiated from an OCRL deleted iPSC line, generated from the isogenic control line NCRM5. Similar observations have been reported for LS stem cell lines generated in an independent recent study (Lo et al., 2024). Our observations strongly suggest that mitochondrial defects noted in cells differentiated from LS patient-derived stem cells arise in cultures containing only neural tissue. Given that the influence of a defective kidney and altered circulating metabolites that could affect mitochondrial function is absent in our model, it seems most likely that the mitochondrial defects seen in OCRL^KO^ NSC and neurons arise from an intrinsic requirement for the function of this gene in these cultured cells.

In this study, we found modest but clear defects in mitochondrial structure and function in NSCs derived from OCRL^KO^ iPSC. These defects persisted when OCRL^KO^ NSCs were differentiated into neurons *in vitro*. In sharp contrast, there were no discernible defects in mitochondrial function in OCRL^KO^ iPSC. These heterogeneities most likely reflect the different levels of the dependency of iPSCs, NSCs, and neurons on the mitochondria as a principal source of energy for their function. Previous studies have reported that stem cells are largely glycolytic in their energy strategy, while NSCs rely more on oxidative phosphorylation, and the dependence of neurons is substantially more on the mitochondria as an energy source (Iwata et al., 2020; Meacham et al., 2022).

Our study of OCRL^KO^ NSC revealed slightly smaller and spherical mitochondria than in controls with reduced branch length, evidence of a moderate level of fragmentation. This presumably results from an imbalance of mitochondrial fission/fusion homeostasis. We also noted that in OCRL^KO^ NSC, MMP was reduced and heterogeneous. Many stem cells are known to be primarily glycolytic, with fragmented mitochondria; fate commitment results in fusion and elongation and a switch to oxidative phosphorylation. It is unclear whether the primary defect in OCRL^KO^ NSC is an imbalance in mitochondrial fusion/fission or if the OCRL function primarily regulates the switch between glycolysis and oxidative phosphorylation. An analysis of the mitochondrial transcriptome revealed only minimal changes, suggesting that alterations in the mitochondrial function may be a consequence of post-transcriptional mechanisms. Surprisingly, we noted in both microscopy and flow cytometry-based assays that there was considerable heterogeneity in MMP in OCRL^KO^ NSC. One possible reason for this heterogeneity may be that in OCRL^KO^ NSCs, there is in fact, a mixture of both neurogenic and gliogenic precursors, in contrast to control NSC cultures, where there are largely only neural precursors (Sharma et al., 2024). The difference in energy metabolism between neural and glial precursor cells is well documented. While neural precursors are plastic and mainly use oxidative phosphorylation, glial precursors mainly depend on glycolysis (Mlody et al., 2016). This heterogeneity in cellular composition most likely also explains our observation that in assays that involve integrated readouts of a large group of cells (e.g., Seahorse-based measurements), there was only a minimal difference between control and OCRL^KO^ NSC.

What is the mechanism by which OCRL regulates mitochondrial structure and function? An immediate possibility is that the elevated levels of PI(4,5)P_2_ in OCRL-depleted cells also impact mitochondrial function. Since we noted that pharmacological inhibition of PIP5K, thereby reducing PI(4,5)P_2_ synthesis, did not rescue MMP in OCRL^KO^ cells, it seems unlikely that the catalytic activity of OCRL or the elevated levels of PI(4,5)P_2_ contribute to the mitochondrial defects described in this study. Presumably, protein–protein interactions of OCRL underlie the mitochondrial phenotype. Interestingly, long-standing biochemical experiments have reported the synthesis of phosphorylated derivatives of phosphatidylinositol in mitochondrial fractions (Hajra et al., 1965; 1968), and an immunolabeling study has suggested a small pool of PI(4,5)P_2_ at the mitochondria (Watt et al., 2002). Given these observations, it remains a possibility that the inhibition of PIP5K was insufficient to reduce PI(4,5)P_2_ levels adequately to rescue the MMP defect in OCRL^KO^ NSC. The reconstitution of OCRL^KO^ with a wild type or catalytically dead OCRL protein will be helpful in resolving this issue more definitively. The OCRL protein itself has not been localized to the mitochondria, and thus its effect on mitochondrial function is most likely indirect (Li et al., 2018; Sheikh-Hamad et al., 2021). One possibility is the role of membrane contact sites involving the mitochondria and other organelles, particularly the endoplasmic reticulum, the main site of phosphatidylinositol biosynthesis. This raises the possibility that OCRL functions at another organelle membrane and may indirectly influence the mitochondria via membrane contact sites, a mechanism that needs further investigation. In summary, our study demonstrates a modest dependence of mitochondrial function on the OCRL protein in tissues where the mitochondria are a principal source of energy production.

## Data Availability

The data presented in the study are deposited in the ArrayExpress repository, accession number E-MTAB-16227.

## Supplementary material

SUPPLEMENTARY FIGURE S1
**(A)** Immunofluorescence images of Control and OCRL^KO^ iPSC-derived NSCs, showing the expression of NSC markers including nestin (orange), SOX2 (green), and nuclei stained with DAPI (cyan). **(B)** Transcriptomic profile showing normalized average expressions of OCRL, NSC, and neuronal genes in control and OCRL^KO^ iPSC-derived NSCs.

SUPPLEMENTARY FIGURE S2
**(A)** Schematic of the experiment to test the ability of the PIP5K inhibitor UNC-3230 on the plasma membrane PI(4,5)P_2_. Representative images showing fields of view from control neural stem cells (NSCs) expressing a plasma membrane-targeted PI(4,5)P_2_ reporter (Akhtar et al., 2021). **(B)** Cells treated with DMSO (vehicle control, VC) display probe localization at the plasma membrane, reflecting baseline PI(4,5)P_2_ levels; **(C,D)** show the effects of treatment with 10 μM and 50 μM UNC-3230, respectively—a selective inhibitor of PIP5K1C—on reporter distribution. Scale bar = 50 μm. **(E)** Quantification of reporter fluorescence at the plasma membrane, reflecting PI(4,5)P_2_ abundance. The Y-axis represents normalized reporter intensity at the plasma membrane, and the X-axis indicates the different treatment conditions.

SUPPLEMENTARY FIGURE S3
**(A)** Immunofluorescence images of Control and OCRL^KO^ iPSC-derived neurons at DIV40, showing the expression of mature neuronal marker, MAP2 (green), and nuclei stained with DAPI (cyan). **(B)** Transcriptomic profile showing normalized average expression of OCRL, NSC, and neuronal genes in control and OCRL^KO^ iPSC-derived neurons.

SUPPLEMENTARY FIGURE S4 Transcriptomic analysis of OCRL^KO^ NSCs shows moderate dysregulation in mitochondrial gene expression. Heatmap showing normalized average expression between OCRL^KO^ NSCs and control of **(A)** GO term: Mitochondrial respiratory chain complex 1 assembly; **(B)** GO term: Mitochondrial translation; **(C)** GO term: Proton motive force driven mitochondrial ATP synthesis.

SUPPLEMENTARY TABLE S1 Table depicting differential gene expression (log2 fold Change) in the OCRLKO NSCs compared to control of 1136 genes localized to the mitochondria from the Human MitoCarta3.0 database. (https://www.broadinstitute.org/files/shared/metabolism/mitocarta/human.mitocarta3.0.html).
