# Supplementary figures and images for: Mitochondrial structure and function in OCRL depleted cells
Source: Front Cell Dev Biol. 2025 Nov 20;13:1679675. doi: 10.3389/fcell.2025.1679675 (PMC12675431; doi:10.3389/fcell.2025.1679675)

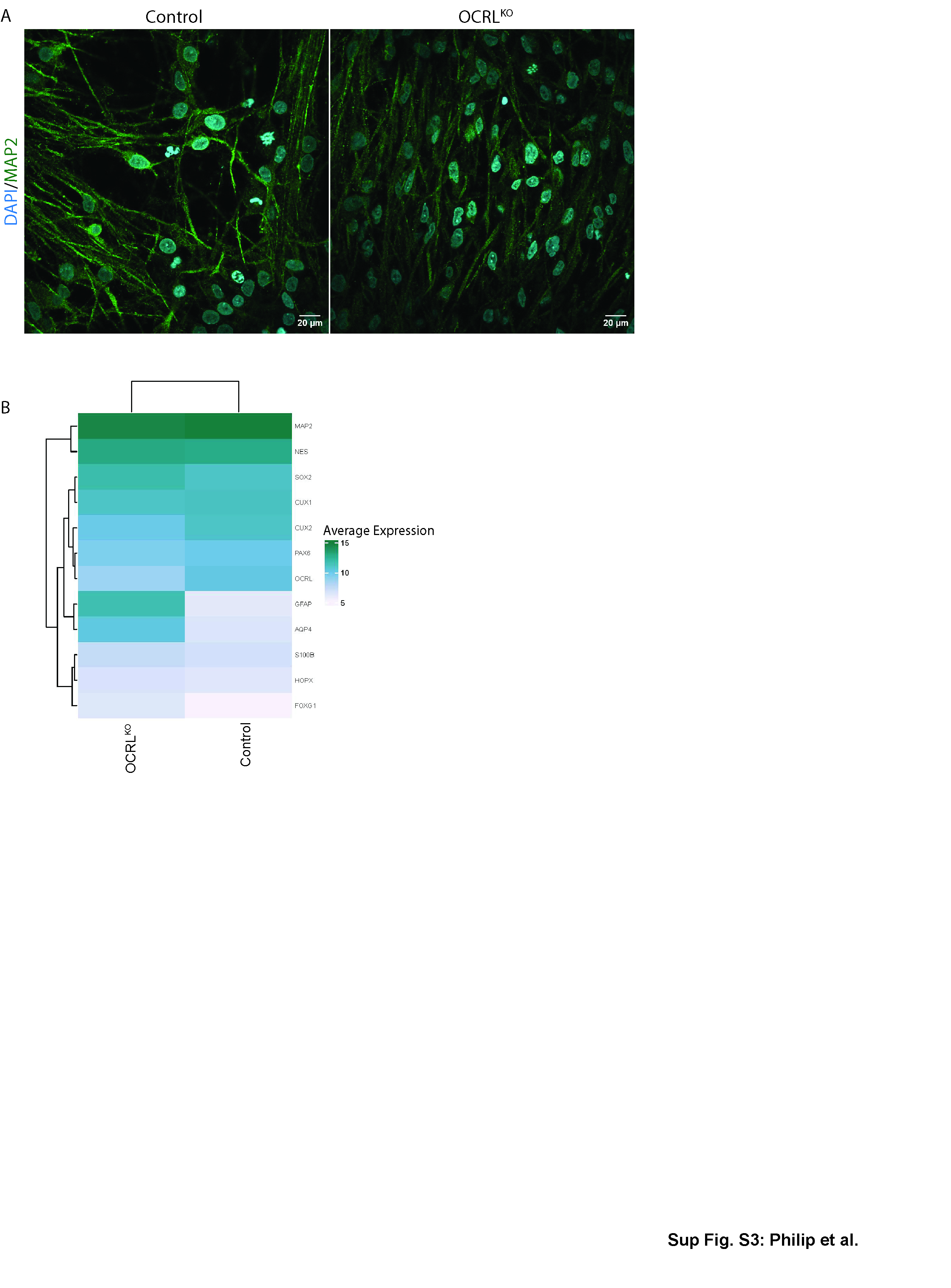

Supplement: Supplementary file 1 [file Image3.tif]

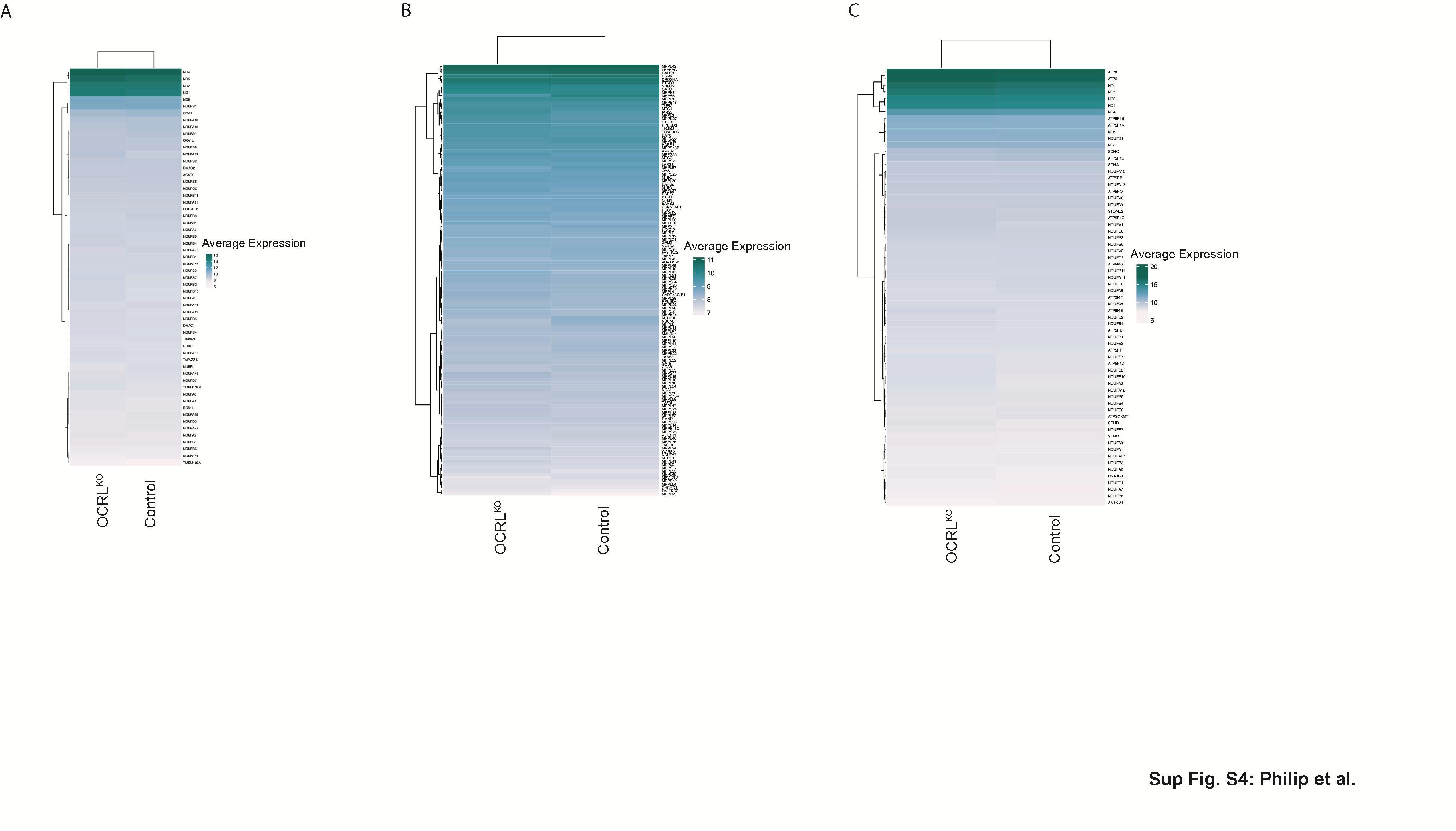

Supplement: Supplementary file 2 [file Image4.tif]

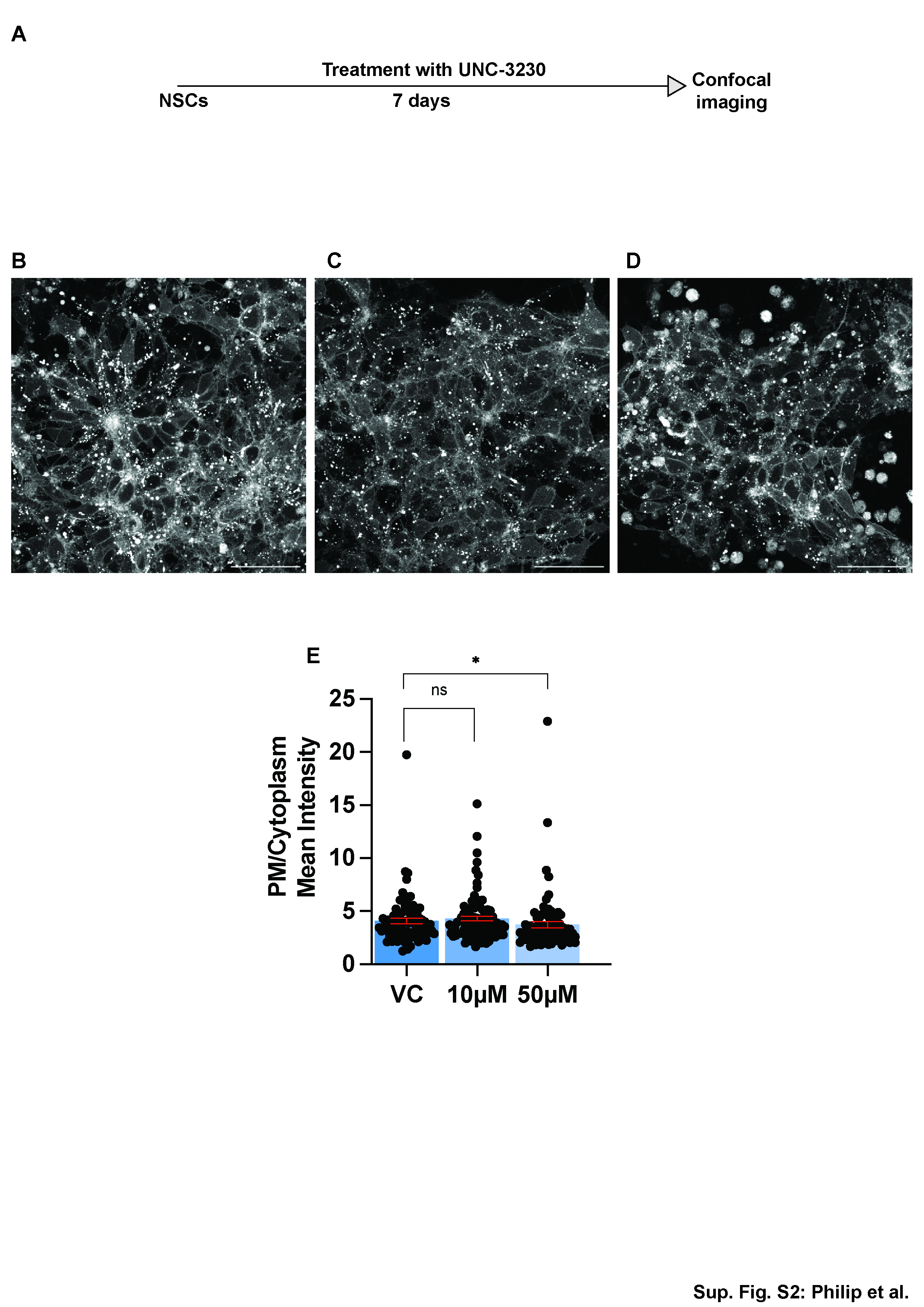

Supplement: Supplementary file 3 [file Image2.tif]

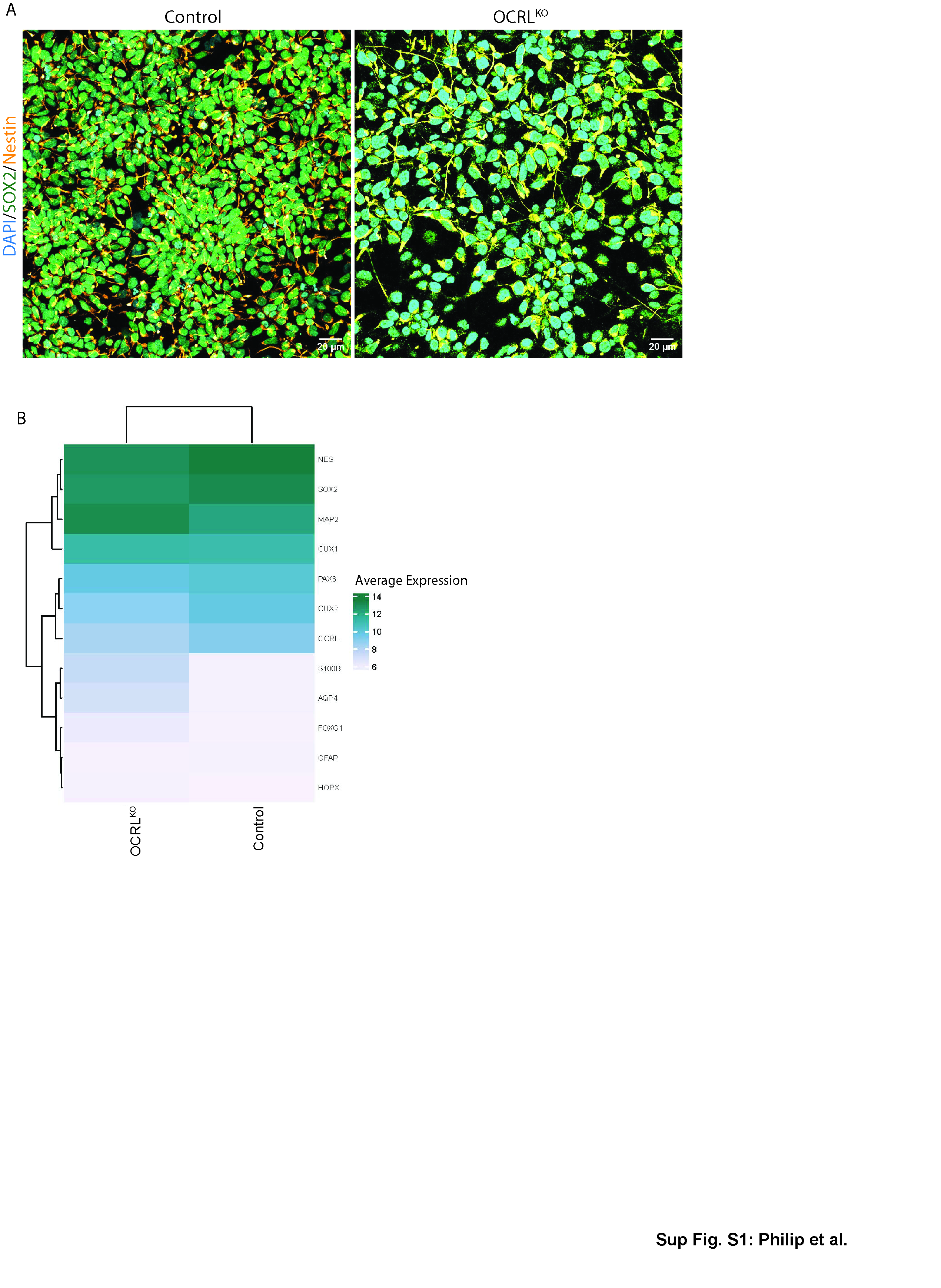

Supplement: Supplementary file 4 [file Image1.tif]
